# Assessing Perception of Wildfires and Related Impacts among Adult Residents of Southern California

**DOI:** 10.3390/ijerph20010815

**Published:** 2023-01-01

**Authors:** Shahir Masri, Erica Anne Shenoi, Dana Rose Garfin, Jun Wu

**Affiliations:** 1 Department of Environmental and Occupational Health, Program in Public Health, University of California, Irvine, CA 92697, USA; 2 Department of Community Health Sciences, Fielding School of Public Health, University of California, Los Angeles, CA 90095, USA

**Keywords:** wildfire, climate change, survey, global warming, risk perception

## Abstract

Major wildfires and their smoke pose a threat to public health and are becoming more frequent in the United States, particularly in California and other populated, fire-prone states. Therefore, it is crucial to understand how California residents view wildfires and engage in risk-reducing behaviors during wildfire events. Currently, there is a knowledge gap concerning this area of inquiry. We disseminated a 40-question cross-sectional survey to explore wildfire perception and knowledge along with related risk-reducing measures and policies among 807 adult residents in the fire-prone region of Orange County, California. Results demonstrated that nearly all (>95%) participants had (or knew someone who had) previously experienced a wildfire. Female gender, knowing a wildfire victim and reporting to have a general interest/passion for environmental issues were the three factors most strongly associated with (1) wildfires (and smoke) being reported as a threat, (2) participants’ willingness to evacuate if threatened by a nearby wildfire, and (3) participants’ willingness to support a wildfire-related tax increase (*p* < 0.05). The majority (57.4%) of participants agreed that the occurrence of wildfires is influenced by climate change, with the most commonly reported risk-reducing actions (by 44% of participants) being informational actions (e.g., tracking the news) rather than self-motivated physical safety actions (e.g., using an air purifier) (29%). The results of this study can help to inform decision- and policy-making regarding future wildfire events as well as allow more targeted and effective public health messaging and intervention measures, in turn helping to reduce the risk associated with future wildfire/smoke episodes.

## 1. Introduction

Climate change has increased the frequency, intensity and spread of major wildfires across many parts of the western United States, in turn posing a threat to public health [1,2,3]. California in particular has experienced some of the greatest wildfire impacts due to a combination of extreme heat waves, high winds and drought coupled with high population densities and encroachment into the wildland-urban interface (WUI) [4,5]. From 1972 to 2018, the annual area burned by wildfires in California increased 5-fold [6]. A 10-fold increase was observed over the last two decades when considering the record-breaking 2020 wildfire year. These deleterious impacts on the California population are widespread: statewide, there were 1081 census tracts housing 5.3 million (13.4%) of California’s 39.5 million residents that experienced wildfires from 2000 to 2020 [7]. Many more residents were additionally exposed to wildfire smoke.

In Southern California, the Santa Ana winds produce a regular wildfire season that peaks in the fall, contributing to some of the most disastrous wildfires in the region [8,9,10]. Numerous public health impacts of these events have been well documented. Wildfire smoke exposure is one of the most readily observable threats, composed of harmful gases and particles associated with both respiratory and cardiovascular disease [11,12,13]. Evidence also links maternal exposure to wildfires during late pregnancy with reduced birth weight and preterm birth [14]. Other impacts include physical and psychological stress related to evacuation, property loss, and physical injury as well as disrupted ecosystems and watersheds such as altered vegetation, habitat availability, and forest fragmentation [15,16].

As wildfires become a growing public health concern in California, it is crucial to evaluate residents’ perceptions and knowledge of wildfire threats and related mitigation strategies to design more effective risk communications and increase adaption, preparation, and support for policies that promote environmental sustainability. To date, studies assessing wildfire perception across the United States are limited. Those that exist have demonstrated differences in perception that have varied both regionally and over time [17,18,19,20], and which mostly focus on perceptions specific to wildfire mitigation and prevention behaviors [19,21,22]. In general, data on wildfire perception among communities in the highly fire-prone state of California are limited, thus limiting our understanding of community risk and exposure [23]. Moreover, limited research exists on how perceptions of wildfire in the context of risk, mitigation behavior, and climate change are associated with demographic factors such as socioeconomics, health status, political affiliation, prior wildfire experience, etc. California residents may vary in their perceptions and knowledge of wildfires as well as the prevention and mitigation actions they might take due to their own socioeconomic status, political affiliations, demographics, and health status.

Previous wildfire experience is important to consider since research suggests that individuals’ perception of natural disasters is associated with prior disaster exposure to such threats [21,24,25,26]. In the case of wildfires, those experiences can influence how one understands and prepares for their occurrence [18]. Compared to firsthand experience, indirect experience of wildfires (e.g., knowing a friend or relative who has been threatened by a wildfire) was not associated with risk-reducing behavior and risk perception of wildfires [27].

In the face of climate change and the emerging public health crisis related to wildfire outbreaks in California, it is essential to better understand wildfire perception and the factors that may influence such perception among residents in fire-prone regions such as Southern California. Herein, we surveyed a demographically diverse sample of residents of Orange County, California in order to understand knowledge and perceptions of wildfires and smoke in the context of risk, precautionary behavior, climate change and policy, and whether responses were associated with demographic factors.

We hypothesized that demographics, such as education, income, and political affiliation, as well as prior wildfire experience, would have a significant effect on residents’ perceptions of wildfire risk, their engagement in related health-protective measures, and their perceptions of a connection between wildfires and climate change. Findings from this research are helpful for policy makers, government agencies and wildfire risk communicators tasked with allocating funds and programs related to wildfire-related adaptation, education and risk-reduction.

## 2. Materials and Methods

### 2.1. Study Population, Recruitment & Survey Design

From 30 January to 14 March 2022, an online cross-sectional survey was administered to adult participants (age ≥ 18) throughout Orange County, California, using the Qualtrics (Provo, UT, USA) survey tool (median survey completion time = 4.9 min). Orange County is among the southern-most coastal counties of California, located between Los Angeles and San Diego. Its population consist of a mix of White (38.5%), Hispanic/Latino (34.1%) and Asian (22.8%) residents, along with smaller fractions of African Americans, Native Americans and other groups, 29.6% of who are foreign-born residents [28]. Those under age 18 account for 21.4% of the population, with just 15.7% being age 65 or older. Of residents who are 25 years old or greater, 41.2% have obtained a bachelor’s degree or higher [28]. The median household income in Orange County is $94,441 (in 2020 dollars), with 9% of residents falling under the poverty line [28].

Recruitment methods included a combination of snowball sampling using local community contacts (e.g., university groups, non-profit organizations, etc.) who helped to circulate the online survey via email listservs and social media platforms (e.g., Facebook, Instagram, etc.). The survey was offered in both English and Spanish (translated by proficient Spanish speaker) to increase inclusivity and maximize representation of the target population given the high proportion of Hispanic/Latino residents who live in the area. As an incentive to enroll in and complete the survey, participants were given the opportunity to enter a raffle that awarded $15 gift certificates to ten randomly selected winners.

Of the surveys opened, 876 (82.6%) were completed, 35 of which were removed for not meeting our age (age ≥ 18) and residential (living in Orange County) inclusion criteria, and another 34 of which were removed owing to unreliable survey completion times (<1.57 min, corresponding to 1st percentile completion time) and likely invalid responses (e.g., checking the first box for all survey answers). Following the exclusion and data cleaning process, a total of 807 surveys remained for analysis.

This study was classified as exempt by the Institutional Review Board (IRB) of the University of California, Irvine, and therefore did not require IRB approval. All survey responses were collected anonymously, and all participants completed the survey only after first providing written informed consent.

### 2.2. Measures

Demographics indicators assessed were gender, age, race/ethnicity, educational attainment, household income, marital status, employment status, number of children, and political affiliation. Health/medical-related measures included “yes/no” questions about chronic disease status (e.g., Do you currently have any chronic diseases?), smoke sensitivity (i.e., Do you consider yourself sensitive to smoke such as smoke from wildfire, cigarette, bonfire, car exhaust?), prior experience of wildfire-related symptoms (i.e., Have you experienced any health symptoms from a wildfire such as coughing, asthma, headaches?) and pregnancy status (i.e., Are you currently pregnant?).

Wildfire-related measures included whether (yes/no) a person had prior experience with a wildfire and whether (yes/no) a person knows someone with such experience (i.e., “Do you know anyone who has either lost their property or life in a wildfire?”). Using a 5-point Likert scale (1 = not at all a threat, 5 = high threat), local and general wildfire measures regarding perception related to life and property (i.e., How much do you view the issue of wildfires in Orange County as a threat to your life and property?, personal health (i.e., How much do you view wildfire smoke as a threat to your health?), and ecosystem health (i.e., How much do you view wildfire smoke as a threat to the health of the ecosystem?). Using a similar 5-point Likert scale (1 = not at all likely, 5 = very likely), participants were also asked about evacuation willingness (How likely are you to evacuate your home/residence if recommended by emergency officials?) and willingness to support a wildfire-related tax increase (i.e., How likely are you to support a tax increase to expand the firefighter workforce and improve access to resources for fire safety?).

Regarding knowledge, participants were asked to self-assess their own perceived wildfire knowledge (i.e., Do you consider yourself knowledgeable about wildfires?) according to a three-option (yes/no/somewhat) response format. As an objective measure of knowledge, participants were then asked to answer a series of 8 questions about wildfires and health effects and rank the extent to which they agree with each statement (1 = strongly disagree, 2 = disagree, 3 = neutral, 4 = agree, 5 = strongly agree). Using the same scale, other measures evaluated whether participants agreed about feeling prepared for wildfire (i.e., I know what to do if there is a wildfire near me) and whether they had resources to keep updated about a wildfire (i.e., I have reliable sources to stay updated on wildfire-related new). Regarding climate change, this same format was applied to evaluate whether they agree that “the occurrence of wildfires is affected by climate change.”

Another measure included self-identification of environmentalism according to a three-option (yes/no/somewhat) response format. (i.e., Would you say you are interested in or passionate about environmental causes?). Finally, using check-all-that-apply format, participants were also asked about their willingness to engage in specific health-protective measures during wildfire episodes (i.e., If you felt at all threatened by a wildfire, which of the following actions would you take?). Options included: Shut off the gas in your home/residence, Buy or use an air cleaner/purifier, Limit your time outdoors, Stay in your home/residence, Listen to emergency officials, Review local air quality reports, and/or Listen to news updates about the fire). A complete list of the question (and answer options) included as outcome variables in the current wildfire survey are presented as Table S1 of the Supplemental Materials.

### 2.3. Data Analysis

All survey responses were recorded by the Qualtrics tool in the form of a.csv file that was subsequently imported and analyzed using SAS software [29]. First, descriptive statistics (e.g., mean, standard deviations) of key outcome variables grouped by key covariates were produced. Outcomes variables included 1-through-5 responses to questions about one’s perception of wildfire and smoke as a threat (1 = “not at all a threat,” 5 = “a major threat”) along with one’s willingness to support policy initiatives and engage in risk-reducing behavior (1 = “not likely,” 5 = “highly likely”). Within these scales, a value of 3 was assigned as “neutral,” indicating perception of neither a threat and/or being supportive or a non-threat and/or being unsupportive. 

Next, bivariate regression models examined the relationship between key predictor variables, covariates, and outcome variables. Multivariate regression analysis subsequently included all statistically significant terms (as determined from bivariate analysis) into individual models (one model for each outcome variable). Covariates included demographic variables as well as characteristics surrounding one’s health status, smoke sensitivity and connection with a prior wildfire victim. Backward stepwise elimination of common non-significant terms (shared by all models) was then applied to result in a more parsimonious model. Statistical significance was set at two-sided *p* = 0.05. 

Lastly, factor analysis was conducted using the “proc factor” tool via SAS software [29]. This method enables the statistical clustering of variables into distinct groups, or factors, based on collinearity. Variables used in our factor analysis included the five Likert scale outcome variables (regarding wildfire threat perceptions and willingness to evacuation and pay more taxes), the eight objective wildfire knowledge questions, along with two additional questions evaluating participants’ wildfire preparedness. In these analyses, we present results from a factor analysis using a three-factor solution, which we believed to be most theoretically reasonable given the particular groups of questions included in our survey (i.e., those relating to wildfire perception and action, general wildfire knowledge, and knowledge on wildfire impacts).

## 3. Results

### 3.1. Descriptive Statistics

Demographics. Of the 807 valid survey responses, 794 and 13 were completed in English and Spanish, respectively. A comparable proportion of male and female participants completed the survey, although slightly more were male (54.8%). The majority of participants (66.8%) reported completing a Bachelor’s Degree or higher, and identified as politically “liberal” (74.5%). The majority of participants also identified as college educated (66.1%), with an income ranging between approximately $50,000 and $100,000 per year (53.9%). See Table 1 for detailed summary statistics. Relative to Orange County as a whole, the current survey pool was made up of slightly more male, fewer White, and more college educated participants. 

Prior exposure and health-related risk factors. As shown in Table 2, 96.0% of respondents reported previous wildfire exposure (e.g., evacuation, smoke exposure, etc.), with 75.1% reporting knowing someone who either lost their life or property in a wildfire. In total, 73.5% reported physical health symptoms from a wildfire (e.g., coughing, asthma, headaches, etc.). The same proportion considered themselves to be sensitive or somewhat sensitive to smoke, whether it be from wildfire smoke, cigarettes, vehicle exhaust, or other combustion sources.

**Figure 1:**
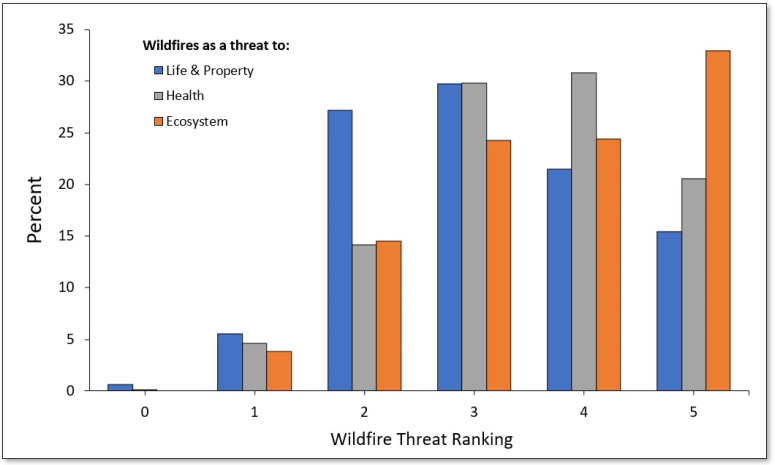
Wildfire threat perception across the sample (0 = no threat, 5 = major threat).

**Figure 2:**
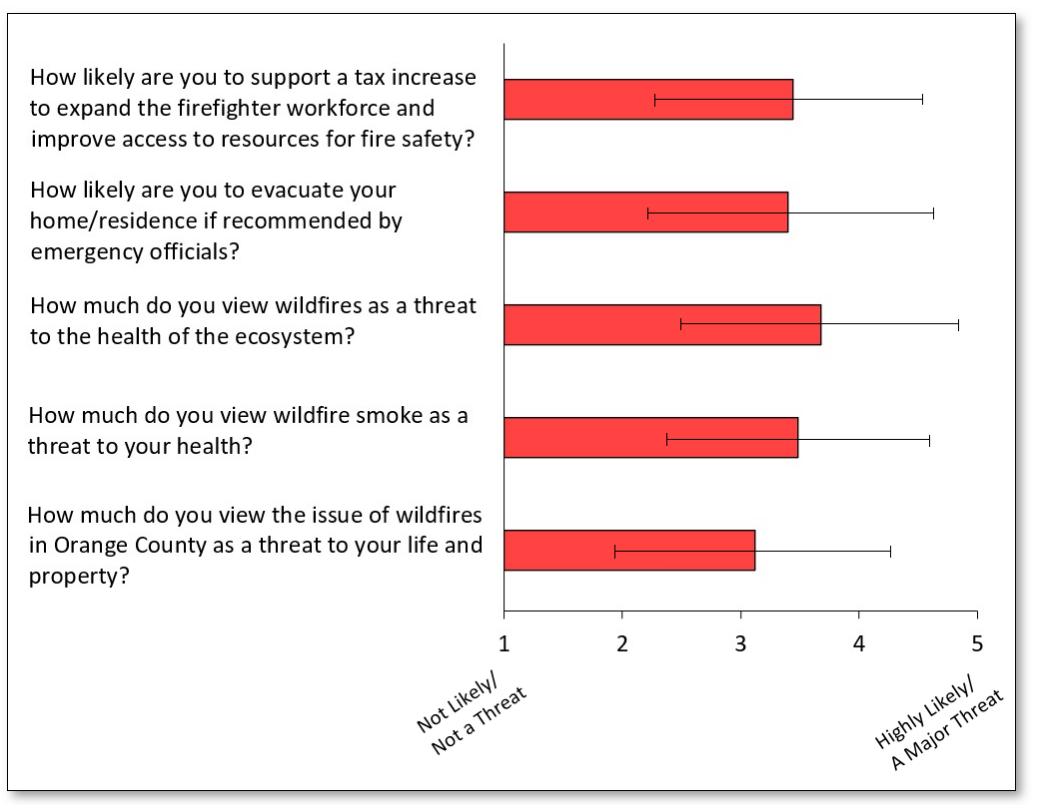
Average response (and standard deviation) among respondents when asked five separate questions (1 = not likely or not at all a threat, 5 = highly likely or a major threat, 3 = neutral).

As shown in Figure 1, the majority of survey respondents ranked wildfire smoke as a threat (response = 4 or 5) to their health (52.0%), with a slightly higher proportion ranking wildfires as a major threat to the ecosystem (58.3%). A lower proportion (36.8%) ranked wildfires as a major threat to their lives and/or property. On average, as shown in Figure 2, the perceived threat of wildfires was ranked between 3 and 4 for threat to life and/or property (M = 3.1, SD = 1.2), health (M = 3.5, SD = 1.1), and ecosystem (M = 3.7, SD = 1.2). 

When asked how likely they would be to evacuate their residence if recommended by emergency officials, less than half (49.0%) were “very likely” to evacuate (response = 4 or 5), with an average likeliness ranking of 3.4 across the survey population. When considering safety and information, less than half (46.4%) of survey respondents reported to “know what to do” in the event of a nearby wildfire, with a similar proportion (48.1%) reporting to be aware of “reliable sources to stay updated on wildfire-related news.”

In considering the implementation of a tax increase to expand the firefighter workforce and improve access to fire safety resources, the majority of respondents were either moderately supportive (45.9%, response = 3 or 4) or highly supportive (48.7%, response = 4 or 5) of such a tax, with only 5.3% being unsupportive. The average likeliness response was 3.5 across the survey population.

**Figure 3:**
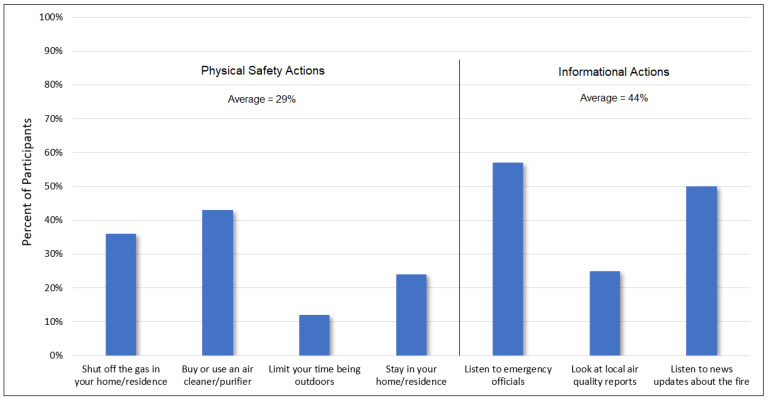
Percent of participants for whom each of the above actions was listed among the “actions you would take if you felt threatened by a wildfire”.

As shown in Figure 3, of the protective actions that participants would take if “feeling threatened by a wildfire,” the most commonly reported action was to listen to emergency officials (57%), followed by listening to news updates (50%) and buying an air cleaner/purifier (43%). The least commonly reported actions included limiting one’s time outdoors (12%) and staying in one’s home/residence (24%). When dividing participants’ actions into two categories (“informational actions” and self-motivated “physical safety actions”), the average response rate was approximately 50% higher for informational actions (44%) compared to physical safety actions (29%).

**Figure 4:**
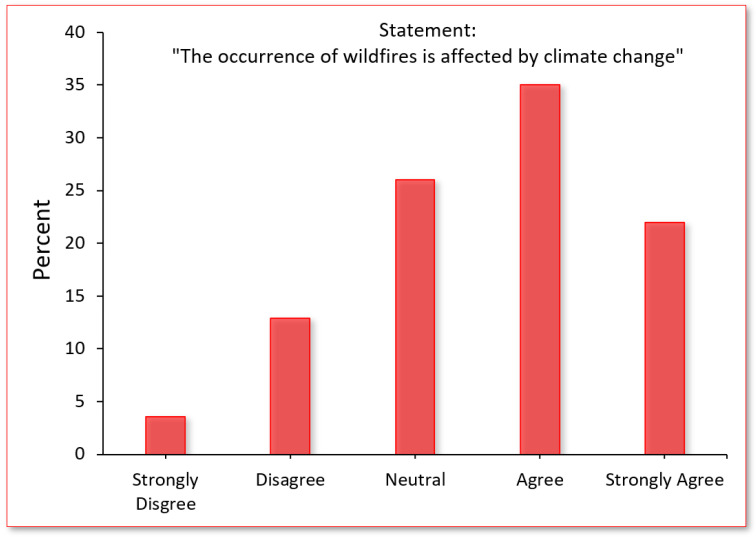
Proportion of survey participants who either agreed or disagreed with statement.

Wildfire-related attitudes. Of respondents (see Figure 4), 57.4% agreed that “the occurrence of wildfires is affected by climate change,” 16.5% disagreed, and 26.1% felt neutral. Of respondents, 54.2% considered themselves knowledgeable about wildfires, while 36.3% and 9.5% considered themselves “somewhat knowledgeable” and “not knowledgeable”, respectively. By comparison, a series of eight wildfire-related questions designed to objectively evaluate one’s wildfire-related knowledge showed only 26.0% of respondents to be knowledgeable about wildfires compared to 61.8% and 12.2% who scored “somewhat knowledgeable” and “not knowledgeable” about wildfires.

### 3.2. Regression Analysis

Table 3 presents multivariate regression analyses of key demographic predictors of wildfire attitudes, beliefs, and support for public policies.

Threat perceptions. Perceptions of wildfires as a threat to life and/or property was associated with gender and race/ethnicity. Women, on average, rated that threat 19% higher than men. Those identifying as Asian/Pacific Islanders and Native Americans rated it 14% (*p* = 0.02) and 20% (*p* < 0.001) lower compared to respondents identifying as Whites. When rating the threat of wildfires to health, each one-unit increase in education level was associated with a 7% increase in perceived threat to health (relative to reference level of “no high school diploma,” *p* < 0.001), whereas identifying as Hispanic/Latino and Native American was associated with a 10% increase (*p* = 0.02) and 26% decrease (*p* < 0.001) in perceive threat, respectively, compared to those identifying as Whites. Finally, regarding wildfire as a threat to the environment, being female was associated with a 15% increase in perceive threat compared to males (*p* < 0.001), while each categorical increase in education level and income was associated with a 7% increase (*p* < 0.001) and 4% decrease (*p* = 0.01) in perceived threat on average, respectively, relative to the reference group. Meanwhile, identifying as Africa American, Asian/Pacific Islander and Hispanic/Latino was associated with 10%, 14%, and 22% increases, respectively, and Native American with a 28% decrease, in perceived threat compared to identifying as White (*p* < 0.02).

When asked one’s likeliness of evacuation if recommended by emergency officials, being female was associated with a 19% increase in reported likeliness of evacuation (*p* < 0.001), while each categorical increase in age and education level was associated with a 5% decrease (*p* = 0.04) and 5% increase (*p* = 0.01) in likeliness of evacuation, respectively, relative to baseline. Similar to previously examined categories, identifying as Hispanic/Latino or Asian/Pacific Islander was associated with an increase, and identifying as Native American a decrease, in likeliness of evacuation (*p* < 0.01).

Similar associations were observed when asking participants about their likeliness of supporting a tax increase to expand the firefighter workforce and improve access to fire safety resources. That is, those identifying as Hispanics/Latinos and Native Americans were generally more supportive and less supportive, respectively, than Whites (*p* < 0.01). While univariate analysis showed older age to be associated with a decrease in support for added taxes, age was no longer a significant covariate once education level was accounted for. Median household income was similarly not significant. Each categorical increase in education level was associated with a 12% increase (*p* < 0.001) in support of such a tax, compared to the reference group. Residents with at least one child were similarly more supportive of a tax increase, as were those who had previously experienced wildfire, who knew a wildfire victim, or who considered themselves to be sensitive to wildfire smoke (*p* < 0.001). When considering the “environmentalist” category, indicating participants’ propensity toward environmental stewardship, such participants tended to rank wildfires and smoke as greater threats, to be more likely to evacuate their residences if recommended by emergency officials, and to be more supportive of a firefighting-related tax increase (*p* < 0.05).

Table 4 presents results from our factor analysis, with moderate-to-high (*r* ≥ 3) positive correlations shown in bold. Three factor categories appeared to demonstrate collinearity in participants responses according to distinct categories that appeared to represent perception and action (Factor 1), general wildfire knowledge (Factor 2), and knowledge on wildfire impacts (Factor 3). Collinearity appeared stronger for Factor 1 variables that involved wildfire perception and action compared to Factor 2, which related mostly to general wildfire knowledge. 

## 4. Discussion

### 4.1. General Findings

This study presents results from a cross-sectional survey on wildfires completed by 807 participants in Orange County, California, through an online interface during the winter and spring of 2022. Results demonstrated that nearly all participants had previously experienced at least some aspect of wildfire (e.g., evacuation, smoke exposure, etc.), with roughly three-fourths reporting to know someone who has either lost their life or property in a wildfire. While prior experience with wildfire was expected given that Southern California is a historically fire-prone area, the high proportion of those who knew victims of wildfire is noteworthy, perhaps reflecting the sharp increase in major wildfires that have been observed across the state in recent decades [30]. What is more, that three-fourths of participants reported to have previously experienced health symptoms and/or sensitivity from wildfire/smoke (e.g., coughing, asthma, headaches, etc.) supports concerns of wildfires as a threat to health while underscoring the importance of wildfire-related health interventions to reduce smoke exposure. 

When examining perceptions of wildfire (or smoke) as a threat, participants tended to assign the highest threat rating to the ecosystem, followed by health and then life/property. While the perceived threat to the ecosystem was expected, the reduced threat reported for smoke may highlight a health-related knowledge gap among the public, in turn suggesting the need for increased public outreach and communication concerning wildfire smoke impacts to health. Nonetheless, on average wildfires (and smoke) were perceived as a moderate-to-high threat among the public, suggesting that such impacts are generally taken seriously. 

When asked how likely they would be to evacuate their residence if recommended by emergency officials, less than half were “very likely” to evacuate. This may potentially reflect the need for increased education surrounding emergency preparedness, a lack of trust in institutions, or limited financial (e.g., inability to afford a hotel) or social (e.g., limited social network to help during evacuation) capacity to evacuate. For instance, while those living outside of the WUI may not view evacuation as a necessity, recent wildfires have underscored that such communities are still highly vulnerable during major wildfires [31,32]. Meanwhile, only about half of participants reported to “know what to do” if threatened by a wildfire and to be aware of “reliable sources to stay updated on wildfire-related news.” Taken together, these findings underscore a high degree of confusion and/or lack of resources surrounding wildfire safety and preparedness, which is important for informing policy surrounding wildfire education and/or access to resources.

Of the protective actions that participants would take if “feeling threatened by a wildfire,” the most commonly reported action was to “listen to emergency officials,” followed by listening to new updates and buying an air cleaner/purifier. That air purifiers were an identified “action” by less than half of the survey population despite being a relatively inexpensive and effective means of reducing indoor PM_2_._5_ concentrations suggests substantial room for improving public health messaging about wildfire smoke and mitigation measures [33,34,35]. Other effective and inexpensive actions to reduce smoke exposure, which were identified by only a quarter or less of the survey population, included limiting one’s time outdoors and staying in one’s home/residence if not in the evacuation zone. In general, “informational actions” were over 50% more readily identified compared to self-motivated “physical safety actions.” This may reflect a limited willingness to compromise one’s behavioral patterns during a wildfire event or simply a lack of prioritization of such measures in the absence of direct guidance by authorities. These findings are similar to those reported by Burke at al. (2021) [36], who found just a 10% increase in the proportion of residents who remained “fully at home” during a heavy wildfire smoke day relative to the mean [36]. Again, however, the current results must be considered in light of the population’s current knowledge of wildfire-related health impacts and the effectiveness of intervention strategies. While this survey focused on urban communities, these findings collectively are important as such communities, while experiencing less direct wildfire burning, often experience evacuation impacts and wildfire smoke.

In considering the implementation of a tax increase to expand the firefighter workforce and improve access to fire safety resources, the overwhelming majority of respondents were supportive of such a tax, while nearly 60% of participants agreed that “the occurrence of wildfires is affected by climate change” (over 3-times more than those who did not agree). Given these complimentary findings, it suggests that residents may be willing to support both wildfire- and climate-related taxes in order to prevent the occurrence of future wildfires. Although this survey did not inquire about one’s willingness to support a carbon-tax or other climate-related tax, prior research from our team indeed showed over 80% of survey respondents to support a carbon tax among a similar demographic [37]. Such support, as seen in Washington State’s Initiative 1631 and 732, however, do not necessarily translate to the adoption of such policies for reasons that are multifaceted and beyond the scope of the present discussion [38,39].

When inquiring about self-reported wildfire knowledge, over half of participants considered themselves knowledgeable about wildfires despite under a third of respondents falling into the “knowledgeable” category according to a series of objective questions designed to evaluate such knowledge. This suggests a potential disconnect between what people know and what they “think” they know, which may influence one’s ability to seek relevant knowledge related to wildfire and/or smoke emergency preparedness. It also highlights an area for public health educators/practitioners to work on.

In general, women reported statistically significant and substantially higher rating of wildfires and/or smoke as a threat and an increase in reported likeliness of evacuation compared to men. Differences between threat perception by males and females may reflect genetic (e.g., higher prevalence of risk-taking behavior among males) and/or societal (e.g., less emergency preparedness among females) [40,41]. Alternatively, or in addition, this may reflect the increased tendency of females to be employed in the healthcare sector (therefore being more aware of related health risks) and/or to be more involved in the at-home caretaking of vulnerable children and elderly (therefore being more concerned with related wildfire/smoke threats) [42,43]. Regardless of the underlying reason, however, this evidence suggests that men may be a demographic on which more emphasis is needed to shift public perception about wildfires as a threat to health.

Similarly, identifying as Hispanic/Latino was associated with an increase in perceived threat, likeliness to evacuate and likeliness to support a wildfire-related tax compared to Whites. This is consistent with national polling that has shown Hispanics/Latinos to be more likely to be “alarmed” or “concerned” about global warming, compared to Whites [44]. In contrast, identifying as Native American was associated with a decrease in perceived wildfire threat and likeliness to support a wildfire-related tax. This may reflect a longer cultural history among Native Americans with the land and in turn greater experience with (and less fear of) wildfires. Alternatively, or in addition, such findings may be due to a lack of understanding surrounding the risks of wildfires and smoke to health and the environment, perhaps reflecting a systemic failure of public health messaging to target this particular group.

A higher education level was generally associated with an increase in perceived threat of wildfires and/or smoke as well as an increased likeliness to evacuate and to support a wildfire tax. These findings are consistent to prior research which has shown a similar socioeconomic relationship between household income and indicators of wildfire concern (e.g., remaining indoors, internet searches of “air filter,”, etc.) [36]. Such results may reflect an increased understanding of the risks associated with wildfire among those with a higher education. Additionally, although income and education are often correlated, it is possible that income renders some homes in more, or less, geographically vulnerable areas. Importantly, residents who knew a wildfire victim or who considered themselves to be sensitive to smoke generally regarded wildfires to be a greater threat compared to other individuals. When considering those categorized as environmentalists, such individuals tended to also regard wildfires and smoke as greater threats, to be more likely to evacuate their residences if recommended by emergency officials, and to be more supportive of a firefighting-related tax increase.

Lastly, through factor analysis, we found collinearity in participants’ responses according to three distinct categories that appeared to represent perception and action (Factor 1), general wildfire knowledge (Factor 2), and knowledge of wildfire impacts (Factor 3). Collinearity appeared strongest for Factor 1, whereas that relating to Factor 2 was more moderate. This is reasonable given our expectation that participants who viewed wildfires a threat according to one metric (e.g., health) would be more likely to view wildfires as a threat according to other metrics (e.g., ecosystem impacts) whereas the same was not expected of Factor 2, which was inherently more objective (assessing general wildfire knowledge). 

### 4.2. Strengths & Limitations

A key strength of this survey is the relatively large number of participants, which enabled us to generate summary statistics concerning each question as well as examine a series of wildfire/smoke-related questions and their potential associations with relevant demographic characteristics through multivariate analysis. Another strength of this study is the evaluation of wildfire-related knowledge in an area that has a long history with seasonal wildfires as well as high variability in socioeconomics. Additionally, relative to most studies which focus only on the direct impact of wildfire burning (e.g., evacuation due to fire), a strength of this study is our added examination of smoke perception and impacts which affects a much larger area and population of residents and results in important health-related indirect impacts. Lastly, this study examined multiple personal and social influential factors such disease status, smoke sensitivity, political affiliation, knowing of prior wildfire victims, and perception of climate change, which lent added insights to our study.

Limitations of this study include the fact that this study is cross-sectional and only represents a snapshot in time. This means that this study cannot account for temporal changes in perception, which may vary by season (e.g., greater concern during wildfire season) or year. Additionally, the participant pool of this survey represented a convenience sample that tended to be comprised predominantly of those who identified as young and middle-aged White/Caucasian individuals who were politically “liberal,” college educated, and earned an annual income ranging between $50,000 and $100,000. Thus, this survey may not necessarily be representative of Orange County (or California) as a whole. Nonetheless, despite this limitation of county- or state-wide representativeness, this demographic is nonetheless common throughout the state and many communities and is therefore relevant to understand. Additionally, questions that assessed participants’ personal firsthand wildfire experience did not include a ranking of severity in exposure, which could be an important aspect to include in future studies. What is more, since the survey was completed on a voluntary basis, it is possible that summary statistics may have been affected by selection bias (e.g., those with prior wildfire experience and/or smoke sensitivity tend to complete the survey at a higher frequency than others).

Additional limitations include the fact that this study was rather urban-focused and therefore did not capture many rural residents who are often characterized by lower educational attainment and lower overall socioeconomic status, and are potentially subjected to greater and/or more frequent wildfire impacts and/or other rural-related occupational exposures. Additionally, to ensure a survey of reasonable length, this study was not able to include all questions that were of interest. For instance, we were not able to assess certain indirect wildfire impacts such as potentially interrupted access to health care, phycological stress, etc. Lastly, given the online (as opposed to in-person) nature of this survey, there is the possibility to have introduced data quality issues (e.g., those unclear about certain questions may have simply checked a box rather than asking a clarifying question). Nevertheless, we expect this influence to be minimal since the wording of each question was carefully selected to ensure clarity. What is more, any potential bias introduced from such error would be expectedly nondifferential.

## 5. Conclusions

This study presents results from a cross-sectional survey on wildfires completed by 807 participants in Orange County, California. Results demonstrated that nearly all participants had previously experienced wildfire in some form, with the majority reporting knowing someone who had either lost their life or property in a wildfire. Identifying as female, knowing a wildfire victim and reporting to have a general interest/passion for environmental issues were the three most influential factors associated with wildfires (and smoke) being considered a threat and with participants’ willingness to both evacuate if threatened by a nearby wildfire and to support a wildfire-related tax increase. The majority of participants agreed that the occurrence of wildfires is influenced by climate change, with the most commonly reported risk-reducing actions tending to be informational actions (e.g., tracking the news) as opposed to physical safety actions (e.g., using an air purifier). Results of this study can help to inform decision- and policy-making regarding future wildfire events as well as allow more targeted and effective public health messaging and intervention measures (e.g., income-based rebates on air purifiers), in turn helping to reduce the risk associated with future wildfire/smoke episodes.

## Figures and Tables

**Table 1:** Characteristics of the sample (*n* = 807).

Race/Ethnicity	%	*n*	Education		
White/Caucasian	59.3	478	No High School Diploma	6.1	49
Latino/Hispanic	12.4	100	High School Diploma	26.4	213
African American	8.6	69	Bachelor’s Degree	54.0	436
Native American	9.2	74	Master’s Degree	10.2	82
Asian/Pacific Islander	9.2	74	Doctoral Degree	2.6	21
Other	1.2	10	Other	0.7	6
**Age**			**Annual Income**		
<18	0.6	5	<$25 K	9.8	73
18–24	17.7	143	$25–50 K	20.7	166
25–39	69.8	563	$50–100 K	53.7	432
40–59	10.2	82	$100–200 K	12.2	98
60+	1.7	14	>$200 K	4.4	35
**Gender**	**%**	***n***			
Male	54.8	441			
Female	44.8	361			
Other	0.3	3			

**Table 2:** Percent of survey population (*n* = 807).

Variable	%
Previous wildfire exposure	96.0
Knows a wildfire victim	75.1
Has previously experienced health symptoms from wildfire	73.5
Is sensitive to smoke	73.6
“Knows what to do” in event of nearby wildfire	46.4
Knows “reliable sources to stay updated on wildfires”	48.1
Knowledgeable about wildfires (self-assessed)	54.2
Knowledgeable about wildfires (objective measure)	26.2
Chronic disease (any)	22.4
Chronic disease (respiratory or cardiovascular)	21.0
Identified as an “environmentalist”	68.3

**Table 3:** Effect estimates (EE) and *p*-values following multivariate regression analysis using key outcome variables.

Parameter	Wildfire as a Threat to Life/Property		Wildfire as a Threat to Human Health		Wildfire as a Threat to the Environment		Likeliness to Evacuate if Recommended		Likeliness to Support Tax for Fire Prevention		Wildfires Influenced by Climate Change	
	E.E.	*p*-Value	E.E.	*p*-Value	E.E.	*p*-Value	E.E.	*p*-Value	E.E.	*p*-Value	E.E.	*p*-Value
**Intercept**	1.44	<0.01	2.29	<0.01	2.25	<0.01	1.77	<0.01	2.24	<0.01	3.71	<0.01
**Gender (Female)**	0.49	<0.01	0.18	0.02	0.51	<0.01	0.53	<0.01	0.06	0.47	0.24	<0.01
**Gender (Male)**	0.00	.	0.00	.	0.00	.	0.00	.	0.00	.	0.00	.
**Age (cat: 1–5) ^a^**	0.08	0.25	−0.05	0.43	0.08	0.27	−0.10	0.20	−0.14	0.05	−0.22	<0.01
**Race (Asian)**	−0.02	0.90	0.43	<0.01	0.58	<0.01	0.43	<0.01	0.13	0.36	0.27	0.06
**Race (African Am.)**	0.13	0.34	0.36	<0.01	0.36	<0.01	0.08	0.57	0.47	<0.01	0.21	0.12
**Race (Hispanic)**	0.11	0.38	0.43	<0.01	0.67	<0.01	0.32	0.01	0.60	<0.01	0.06	0.60
**Ethnicity (Native Am.)**	−0.21	0.17	−0.53	<0.01	−0.73	<0.01	−0.70	<0.01	−0.35	0.01	−0.49	<0.01
**Ethnicity (Caucasian)**	0.00	.	0.00	.	0.00	.	0.00	.	0.00	.	0.00	.
**Education (cat: 1–5) ^b^**	−0.05	0.34	0.11	0.022	0.09	0.056	0.07	0.16	0.29	<0.01	0.05	0.36
**Income (cat: 1–5) ^c^**	0.08	0.10	−0.02	0.72	−0.07	0.14	0.08	0.09	−0.04	0.33	0.12	<0.01
**# Children (cat: 1–4) ^d^**	0.09	0.31	0.24	<0.01	−0.04	0.62	−0.06	0.51	0.32	<0.01	−0.39	<0.01
**Chronic Disease ^e^**											.	.
**No**	0.24	0.02	0.06	0.54	0.20	0.05	0.33	<0.01	0.23	0.02	0.20	0.06
**Yes**	0.00	.	0.00	.	0.00	.	0.00	.	0.00	.	0.00	.
**Environmentalist (1–3) ^f^**	0.52	<0.01	0.38	<0.01	0.55	<0.01	0.48	<0.01	0.34	<0.01	0.05	0.31
**Knows wildfire victim**											.	.
**No**	−0.82	<0.01	−0.61	<0.01	−0.62	<0.01	−0.22	0.04	−0.56	<0.01	0.14	0.20
**Yes**	0.00	.	0.00	.	0.00	.	0.00	.	0.00	.	0.00	.
**Smoke Sensitive (1–3)**	0.16	<0.01	0.12	<0.01	−0.06	0.17	0.14	<0.01	0.01	0.81	0.06	0.23

^a^ Age categories: 1 (<18 years), 2 (18–24 years), 3 (25–39 years), 4 (40–59 years), and 5 (≥60 years); ^b^ Educational categories: 1 (no high school completion), 2 (high school completion), 3 (college completion), 4 (earned master’s-level degree), and 5 (earned doctoral degree); ^c^ Annual household income categories: 1 (<$25,000), 2 ($25,000–$50,000), 3 ($50,000–$100,000), 4 ($100,000–$200,000), and 5 (>$200,000); ^d^ Number of children categories: 1 (no children), 2 (1 child), 3 (2–4 children), and 4 (>4 children); ^e^ Chronic disease referred only respiratory and cardiovascular diseases. ^f^ Environmentalist categories: 1 (no), 2 (somewhat), and 3 (yes) when responding to question that asked: “Would you say you are interested in or passionate about environmental causes?”.

**Table 4:** Results of factor analysis.

	Rotated Factor Pattern		
	Factor 1	Factor 2	Factor 3
Degree of self-reported environmentalism	**0.39**	0.15	0.06
Threat to property	**0.77**	0.10	0.00
Threat to health	**0.80**	0.09	0.00
Threat to environment	**0.77**	0.13	−0.14
Likeliness to evacuate	**0.71**	0.30	−0.21
Support fire tax	**0.65**	0.04	0.13
Wildfires occurrence changing	0.11	**0.57**	0.09
Wildfires duration changing	0.04	**0.51**	0.15
Climate influencing wildfires	0.07	**0.56**	−0.09
Smoke affects people the same	0.15	**0.59**	−0.19
Every person has the same sensitivity to wildfire smoke.	0.16	0.07	**0.46**
Wildfires have ecological benefits.	−0.10	0.26	**0.42**
Wild animals are not affected by wildfires.	−0.13	−0.10	**0.71**
I know what to do if there is a wildfire near me.	0.13	**0.40**	0.14
I have reliable sources to stay updated on wildfire-related news.	0.20	**0.34**	0.14

Note: Bold indicates moderate-to-high (*r* ≥ 3) positive correlation.

## Data Availability

Data used in this study will be made available upon request from the authors.

## Supplementary Materials

The following supporting information can be downloaded at: https://www.mdpi.com/article/10.3390/ijerph20010815/s1, Table S1. All questions (and answer options) included as outcome variables in wildfire survey.
