# Supplementary material for: Assessing Perception of Wildfires and Related Impacts among Adult Residents of Southern California
Source: Int J Environ Res Public Health. 2023 Jan 1;20(1):815. doi: 10.3390/ijerph20010815 (PMC9820212; doi:10.3390/ijerph20010815)
Supplement: Supplementary file 1 [file ijerph-20-00815-s001.zip › ijerph-2098725-supplementary.pdf]

Supplemental Materials

Table S1. All questions (and answer options) included as outcome variables in wildfire survey.

Do you currently have any chronic diseases? • Yes • No
Do you currently have a heart or lung disease (including heart failure, angina, ischemic heart disease, chronic obstructive pulmonary disease, emphysema or asthma)? • Yes • No
Are you currently pregnant? • Yes • No
Would you say that you are interested in or passionate about environmental causes? • Yes • No • Somewhat
Have you experienced a wildfire before (including smelling smoke, seeing any part of the fire, evacuating or taking another action in response to a wildfire)? • Yes • No
Do you know anyone who has experienced a wildfire before (including smelling smoke, seeing any part of the fire, evacuating or another action in response to a wildfire)? • Yes • No
Do you know anyone who has either lost their property or life in a wildfire? • Yes • No
Have you experienced any health symptoms from a wildfire (such as coughing, asthma, headaches)? • Yes • No
Do you consider yourself sensitive to smoke (such as smoke from wildfire, cigarette, bonfire, car exhaust)? • Yes • No • Somewhat
On a scale of 1 to 5 (1=not at all, 5=a lot), how much do you view the issue of wildfires in Orange County as a threat to your life and property?
On a scale of 1 to 5 (1=not at all, 5=a lot), how much do you view wildfire smoke as a threat to your health?
On a scale of 1 to 5 (1=not at all, 5=a lot), how much do you view wildfires as a threat to the health of the ecosystem?
If you felt at all threatened by a wildfire, which of the following actions would you take? (Select all that apply): (answer options already presented in Figure 3 of main text)
Of the protective actions you would take during a wildfire, at what distance away from the wildfire would you feel the need to take such actions? • < 5 miles • 5-15 miles • 15-30 miles • > 30 miles
On a scale of 1 to 5 (1=not at all, 5=a lot), how likely are you to evacuate your home/residence if recommended by emergency officials?
On a scale of 1 to 5 (1=not at all, 5=a lot), how likely are you to support a tax increase to expand the firefighter workforce and improve access to resources for fire safety?
Do you consider yourself knowledgeable about wildfires? • Yes • No • Somewhat
Please rate the extent to which you agree/disagree with the following statement (1 = Strongly Disagree, 2 = Disagree, 3 = Neutral, 4 = Agree, 5 = Strongly Agree): The occurrence of wildfires in Orange County has increased over the past decade.
Please rate the extent to which you agree/disagree with the following statement (1 = Strongly Disagree, 2 = Disagree, 3 = Neutral, 4 = Agree, 5 = Strongly Agree): The duration of wildfires in Orange County has increased over the past decade.
Please rate the extent to which you agree/disagree with the following statement (1 = Strongly Disagree, 2 = Disagree, 3 = Neutral, 4 = Agree, 5 = Strongly Agree): The occurrence of wildfires is affected by climate change.
Please rate the extent to which you agree/disagree with the following statement (1 = Strongly Disagree, 2 = Disagree, 3 = Neutral, 4 = Agree, 5 = Strongly Agree): Wildfire smoke can affect any person's health.
Please rate the extent to which you agree/disagree with the following statement (1 = Strongly Disagree, 2 = Disagree, 3 = Neutral, 4 = Agree, 5 = Strongly Agree): Every person has the same sensitivity to wildfire smoke.
Please rate the extent to which you agree/disagree with the following statement (1 = Strongly Disagree, 2 = Disagree, 3 = Neutral, 4 = Agree, 5 = Strongly Agree): Wildfires have ecological benefits.
Please rate the extent to which you agree/disagree with the following statement (1 = Strongly Disagree, 2 = Disagree, 3 = Neutral, 4 = Agree, 5 = Strongly Agree): Wild animals are not affected by wildfires.
Please rate the extent to which you agree/disagree with the following statement (1 = Strongly Disagree, 2 = Disagree, 3 = Neutral, 4 = Agree, 5 = Strongly Agree): I know what to do if there is a wildfire near me.
Please rate the extent to which you agree/disagree with the following statement (1 = Strongly Disagree, 2 = Disagree, 3 = Neutral, 4 = Agree, 5 = Strongly Agree): I have reliable sources to stay updated on wildfire-related news.
